# First-Cut Yield and Forage Quality of Grass–Legume Mixtures Differing in Composition and Intended Use Across Two Years

**DOI:** 10.3390/plants15121909

**Published:** 2026-06-19

**Authors:** Gabrielė Gvazdauskė, Vilma Kemešytė, Gintarė Šidlauskaitė, Žydrė Kadžiulienė, Kristina Jaškūnė

**Affiliations:** Lithuanian Research Centre for Agriculture and Forestry, Institute of Agriculture, Kedainiai District, LT-58344 Akademija, Lithuania; vilma.kemesyte@lammc.lt (V.K.); gintare.sidlauskaite@lammc.lt (G.Š.); zydre.kadziuliene@lammc.lt (Ž.K.); kristina.jaskune@lammc.lt (K.J.)

**Keywords:** grass–legume mixtures, dry matter yield, forage quality, mixture type, botanical composition, year-to-year variation

## Abstract

Grass–legume mixtures can improve forage productivity and quality, but their performance may vary between years. This study evaluated grass–legume mixture types differing in composition and intended use (forage, universal, and grazing) across two growing seasons, focusing on the first cut. Mixtures were assessed for dry matter yield, botanical composition, chemical composition, and plant height, and principal component analysis (PCA) was used to describe multivariate trait patterns. The year strongly affected mixture performance. Mean dry matter yield decreased from 6949 ± 212 kg ha^−1^ in 2023 to 1588 ± 94 kg ha^−1^ in 2024, corresponding to an approximately 76% reduction. In 2023, forage mixtures produced the highest yield, followed by universal and grazing mixtures, whereas in 2024, differences among mixture types were not significant. Botanical composition shifted toward a higher legume proportion in 2024, while mixture type differences in chemical composition largely converged, except for DMD. PCA showed clearer separation among mixture types in 2023, with PC1 and PC2 explaining 64.9% and 17.6% of variance, respectively, whereas separation weakened in 2024. These results indicate that mixture composition and intended use influenced productivity and quality mainly under more favorable growing conditions, while year-to-year variation strongly constrained first-cut mixture performance.

## 1. Introduction

Sustainable feed production has become an increasingly important goal in grassland-based agriculture, particularly because conventional grassland management often relies on practices with low nitrogen use efficiency that may negatively affect soil and water systems [1]. These limitations reduce the sustainability of agricultural production, increase environmental pressure, and threaten the long-term ecological viability of these systems [2]. Beyond environmental considerations, many traditional grasslands are characterized by low species richness and dominated by a small number of grass species, which may limit the stability of biomass production under variable environmental conditions [3,4]. With increasing climatic variability, such species-poor systems may become more prone to fluctuations in plant growth, nutrient uptake, and yield, thereby reducing the resilience of grass-based feed production [5]. 

Grass–legume mixtures are considered an alternative to grass monocultures for improving the sustainability and productivity of grasslands, particularly under increasing climatic variability. Their benefits are largely associated with the inclusion of legumes, which can reduce the need for synthetic nitrogen fertilization through biological nitrogen fixation and contribute to improved soil properties and overall ecosystem functioning [6,7]. In terms of productivity, mixtures have often been shown to achieve comparable or higher yields than grass monocultures, especially under low nitrogen inputs, due to complementary resource use and biological nitrogen fixation [6,8,9]. Moreover, they provide more stable forage production under variable growing conditions [10]. Improved water use efficiency and drought tolerance in mixtures are often linked to greater root surface area and depth, which facilitate access to deeper soil water and support ecosystem resilience under extreme climatic events [11,12].

The ability of grass–legume mixtures to stabilize productivity and maintain forage quality is strongly influenced by functional diversity, which is a key mechanism underpinning efficient grassland functioning [13]. Species that differ in growth strategy, phenology, and nitrogen acquisition can modify competitive and complementary interactions, thereby affecting growth dynamics and nitrogen accumulation in plant biomass, resulting in variability of chemical composition and digestibility [8,14]. However, the effects of functional diversity are not universal and depend strongly on the selected grass and legume species, as well as on their proportions in the mixtures [15]. In particular, legumes often increase the leaf:stem ratio, thereby improving forage digestibility [16]. Moreover, grass–legume mixtures typically have higher nitrogen concentrations in aboveground biomass, which contributes to higher crude protein content and enhanced nutritive value [17].

While productivity and climate resilience are key advantages of grass–legume mixtures, their successful implementation in livestock production systems ultimately depends on forage quality attributes relevant to animal nutrition. Chemical composition traits, including crude protein concentration, neutral detergent fiber (NDF), acid detergent fiber (ADF), and digestibility, play a critical role in livestock production by affecting nutrient utilization efficiency and associated emission outputs [18,19]. In addition to management and species composition, climatic conditions strongly regulate forage chemical composition. Temperature, water availability and drought stress can influence C and N allocation within plant tissues and thus affect crude protein content, fiber fractions and digestibility [20]. These effects may occur directly through plant metabolic processes or indirectly through changes in growth, phenological development, and tissue properties [21,22]. Therefore, variation in temperature and precipitation may affect both the average value and consistency of forage quality. Under variable climatic conditions, the main challenge is not only to produce high biomass but also to maintain stable yield and forage quality over time.

This study aimed to determine how grass–legume mixture types, defined by both botanical composition and intended use, influence first-cut dry matter yield, nutritive value, and year-to-year variation across two growing seasons. The first cut was emphasized because it is typically the most productive harvest and can account for up to approximately 50% of the total annual yield, while also providing forage with high nutritive value [23]. Therefore, focusing on the first cut allows assessment of the productivity and forage quality potential of grass–legume mixtures under practical forage production conditions. 

## 2. Results

### 2.1. Dry Matter Yield

**Figure 1:**
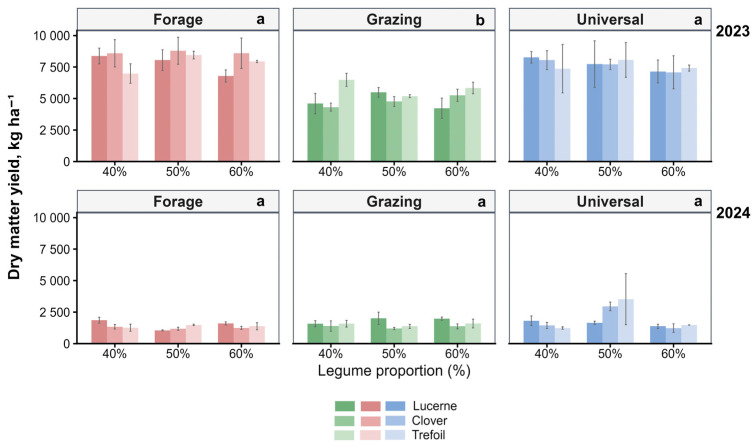
Dry matter yield of grass–legume mixtures at the first cut. Different letters indicate significant differences between mixture types (*p* < 0.05).

**Figure 2:**
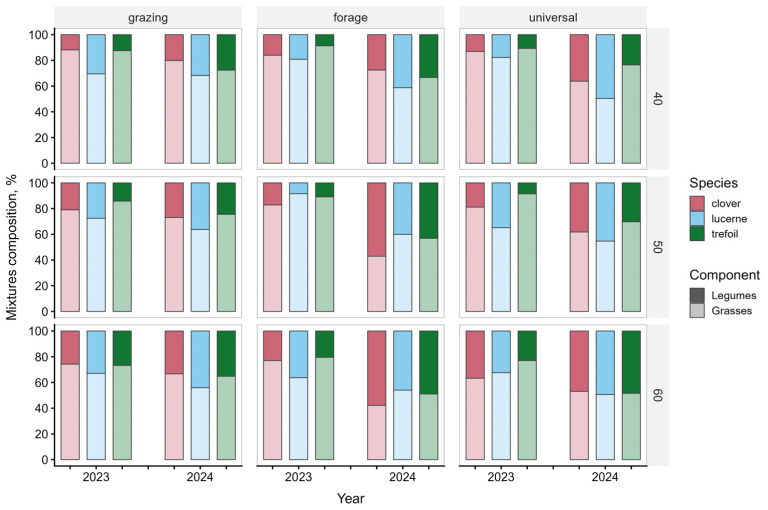
Botanical composition of grass–legume mixtures at the first cut.

In the first cut of 2023, the mean dry matter yield across all treatments was 6949 ± 212 kg ha^−1^ (mean ± SE, *n* = 81), ranging from 3235 to 11,217 kg ha^−1^ (Figure 1). The linear mixed-effects model showed significant effects of mixture type (*F* = 17.74, *p* < 0.0001), year (*F* = 142.55, *p* < 0.0001), and the mixture type × year interaction (*F* = 8.92, *p* = 0.0004) on dry matter yield. Forage mixtures produced the highest yield (8066 ± 259 kg ha^−1^), followed by universal mixtures (7650 ± 337 kg ha^−1^), whereas grazing mixtures showed markedly lower yields (5132 ± 198 kg ha^−1^). Tukey-adjusted pairwise comparisons showed that forage and universal mixtures produced significantly higher yields than grazing mixtures in 2023, whereas forage and universal mixtures did not differ significantly from each other. The yield difference between forage and grazing mixtures reached approximately 37%, demonstrating a clear effect of mixture type on dry matter productivity. This pattern was largely consistent with the botanical composition of the mixtures: forage mixtures were strongly grass-dominated (82.2% grasses and 17.8% legumes), whereas grazing mixtures contained a slightly lower proportion of grass (77.5%) and a higher proportion of legumes (22.5%), which corresponded with their reduced dry matter yield (Figure 2). In contrast, legume species in mixtures had no significant effect on yield (*p* = 0.751). Although the differences were not statistically significant, mixtures containing birdsfoot trefoil had a numerically higher mean yield (7083 ± 313 kg ha^−1^), followed by mixtures with clover (7020 ± 406 kg ha^−1^) and lucerne (6745 ± 385 kg ha^−1^).

The effect of the year was highly significant (*p* < 0.001), with total dry matter yield in 2024 being approximately 76% lower than in 2023. In 2024, the mean dry matter yield was 1588 ± 94 kg ha^−1^ (mean ± SE, *n* = 78), with values ranging from 672 to 7576 kg ha^−1^. During the first cut of 2024, yields remained uniformly low across all mixture types. The highest mean yield was obtained in the universal mixtures (1827 ± 264 kg ha^−1^), followed by grazing (1573 ± 92 kg ha^−1^) and forage mixtures (1380 ± 67 kg ha^−1^). Despite these numerical differences, Tukey-adjusted pairwise comparisons indicated no significant differences among mixture types in 2024, showing that the reduced yields were consistent across all mixture types under the unfavorable growing conditions of that year. Among the legume groups, mixtures with trefoil exhibited the highest mean dry matter yield (1664 ± 246 kg ha^−1^), followed closely by lucerne (1658 ± 90 kg ha^−1^) and clover mixtures (1433 ± 111 kg ha^−1^). Nevertheless, these differences were minor and statistically non-significant, indicating comparable yield performance across legume species.

Furthermore, legume proportion had no significant effect on dry matter yield (*p* = 0.475). Thus, variation in dry matter production was associated mainly with year, mixture type, and their interaction.

### 2.2. Botanical Composition

In 2023, the botanical composition differed slightly among mixture types but was consistently dominated by grasses (Figure 2). Forbs and other non-sown species accounted for only a minor proportion of first-cut biomass in both years, averaging 0.7% in 2023 and 0.4% in 2024. Therefore, they were not presented separately in Figure 2, and the botanical composition results focus on the grass–legume balance. Grazing mixtures consisted of 77.5% grasses and 22.5% legumes, while forage mixtures contained 82.2% grasses and 17.8% legumes. In universal mixtures, grasses accounted for 78.3% and legumes for 21.7%. In 2024, the proportion of legumes increased across all mixture types. In grazing mixtures, the legume share rose to 31.0%, accompanied by a decrease in grasses to 69.0%. Forage mixtures showed an even stronger shift, with legumes increasing to 43.9% and grasses decreasing to 56.1%. In universal mixtures, legumes comprised 40.8% and grasses 59.2%. Overall, the comparison between 2023 and 2024 showed a clear trend: in all three mixture types, the proportion of legumes increased markedly, while the share of grasses declined accordingly. These shifts were statistically significant across all legume species and mixture types (*p* < 0.01), indicating a strong and consistent year effect. In 2023, legume proportion differed significantly among species, with trefoil showing a lower contribution than lucerne. In contrast, in 2024, significant differences were observed among mixture types, with forage mixtures exhibiting a higher legume proportion than grazing mixtures.

### 2.3. Forage Chemical Composition

Mixture type had a significant effect on most chemical composition traits in 2023 (Table 1). A significant mixture type × year interaction was detected for CP, CF, MADF, NDF, and WSC (*p* < 0.0001), indicating that treatment effects differed between years. Among all types, grazing mixtures showed the clearest and strongest differences. They exhibited the highest nutritive value, with substantially higher crude protein concentrations, lower fiber fractions, and higher dry matter digestibility than universal mixtures. In 2023, grazing mixtures had the highest CP and WSC levels and the lowest CF, MADF and NDF values across all mixture types. Forage and universal mixtures showed broadly similar chemical composition profiles, with higher fiber fractions and lower CP and WSC than grazing mixtures. For DMD, grazing mixtures had the highest values, universal mixtures the lowest, and forage mixtures showed intermediate values. The magnitude of these differences was substantial in 2023, particularly for NDF and WSC, where differences between grazing and forage mixtures reached approximately 9 and 12 percentage units, respectively.

**Figure 3:**
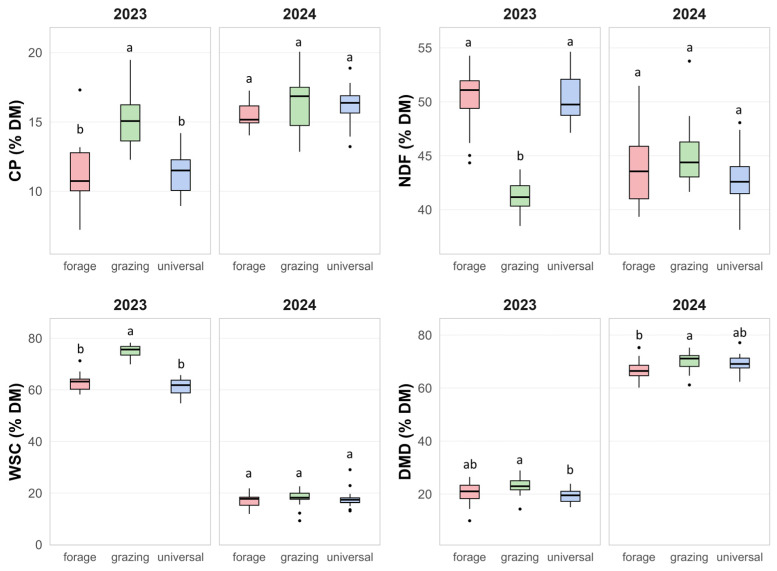
Crude protein (CP), neutral detergent fiber (NDF), water-soluble carbohydrates (WSC), and dry matter digestibility (DMD) of grass–legume mixtures in 2023 and 2024. Different letters indicate significant differences among mixture types within each year (Tukey-adjusted pairwise comparisons, *p* < 0.05).

In 2024, the effect of mixture type weakened, and no statistically significant differences were found for most chemical composition traits (*p* > 0.05) (Table 1; Figure 3). Mean values of CP, CF, MADF, NDF, and WSC were nearly identical across all mixture types, indicating a marked reduction in compositional differentiation compared with 2023. DMD still differed among mixture types in 2024, with grazing mixtures showing higher digestibility than forage mixtures, while universal mixtures had intermediate values. Year had a significant main effect on all chemical composition traits (*p* < 0.0001), with generally higher crude protein concentrations and slightly lower fiber fractions in 2024 compared with 2023. Overall, the chemical composition results showed that mixture type differences were strongly expressed in 2023, whereas in 2024 they largely converged, except for DMD.

### 2.4. Principal Component Analysis

**Figure 4:**
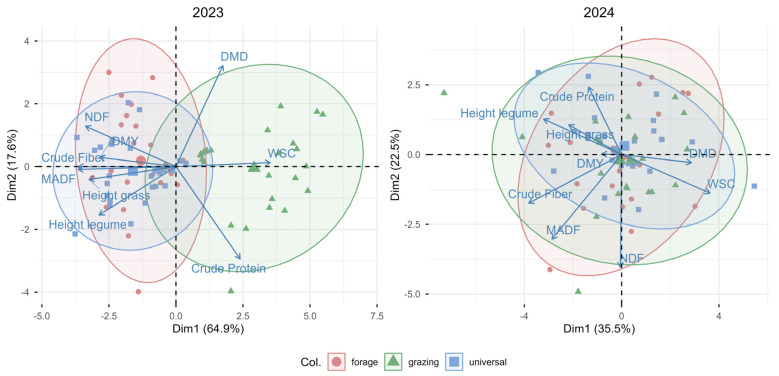
PCA of mixtures based on first-cut yield, plant height, and chemical composition traits.

To further explore the multivariate structure of mixture traits and their combined contribution to differences among mixture types, a principal component analysis (PCA) was performed (Figure 4). The analysis included variables describing chemical composition traits, together with first-cut dry matter yield and plant height. In 2023, the first two principal components explained a high proportion of the total variance in the data, with PC1 explaining 64.9% and PC2 17.6%. Together, these components accounted for almost 83% of the total variance and indicated a clear multivariate structure of traits during that year. In 2023, PC1 mainly separated productivity- and structure-related traits from nutritive value traits. DMY, plant height, CF, MADF, and NDF were associated with one side of the axis, whereas CP, WSC, and DMD were associated with the opposite side, indicating a trade-off between biomass accumulation and nutritive value. Mixture types showed clearer visual separation along the main PCA axis, indicating distinct multivariate trait patterns related to yield, plant height, and chemical composition traits. In 2024, the proportion of variance explained by PC1 decreased to 35.5%, while PC2 explained 22.5% of the variance, and overlap among mixture types increased. In contrast to 2023, DMY was less strongly associated with the main multivariate gradient in 2024, and variation was more strongly associated with chemical composition traits, particularly WSC, DMD, MADF, NDF, and CP. This pattern suggested a weaker multivariate structure and less distinct visual separation among mixture types compared with 2023. Nevertheless, the ordination pattern indicated that grazing mixtures maintained a comparatively consistent position between two years, whereas forage and universal mixtures showed greater dispersion and visual overlap in 2024.

## 3. Discussion

The multivariate patterns, as revealed by principal component analysis (PCA), integrated yield, plant height, and chemical composition traits, providing insight into trait responses across the two study years. In 2023, the clear separation of mixture types along a productivity-quality gradient reflected strong multivariate trait contrasts among mixtures, consistent with a classic trade-off between biomass accumulation and nutritive value [24,25]. The compression of this gradient in 2024 suggests that the range of trait expression was constrained under the conditions of that year, thereby narrowing multivariate trait differentiation among mixture types. Notably, grazing mixtures retained a relatively consistent multivariate position between the two years. This may reflect their compositional characteristics and orientation toward higher nutritive value, partly associated with the inclusion of white clover in some grazing mixtures. White clover is known to increase crude protein concentration and improve nutritive value [26,27]. The observed mixture type effects should be interpreted as effects of compositional mixture categories designed for different intended uses, rather than as effects of mixture type independent of species composition. Such compositional effects are closely linked to the deliberate selection of species adapted to temperate grassland systems in Northern Europe. In these systems, stress-tolerant grasses such as tall fescue and productive hybrids such as *×Festulolium* are combined to exploit complementary agronomic and compositional traits. The high productivity and favorable nutritive value of *×Festulolium*, together with the greater tolerance of tall fescue to drought or excess moisture, may support mixture performance under variable growing conditions [28]. This compositional complementarity is a key principle underlying mixture design and may partly explain the relatively consistent positioning of grazing mixtures across contrasting years. In contrast, forage and universal mixtures showed greater shifts between the two study years, suggesting that mixtures designed primarily to maximize structural biomass production may be more sensitive to annual growing conditions than those oriented toward nutritive value. Such responses likely reflect differences in mixture composition and strategies of resource use among mixture types, indicating a trade-off between high productivity under favorable conditions and more consistent trait expression under less favorable conditions.

Consistent with these multivariate patterns, year-to-year variation was the main factor associated with mixture performance during the study period. Differences between years far exceeded those among mixture types across all measured parameters, particularly in biomass production. The strong year effect suggests that seasonal growing conditions may override compositional differences among mixtures, limiting the extent to which mixture composition can influence productivity. Under the more favorable conditions of 2023, a clear yield ranking was observed among mixture types: forage > universal > grazing. This ranking reflected their intended mixture design, with forage mixtures oriented toward biomass production, universal mixtures representing a balance between yield and quality, and grazing mixtures prioritizing nutritive value. However, these differences were not observed in 2024, when biomass production sharply declined. Because soil water status and plant stress indicators were not directly quantified, this decline should not be attributed to climatic stress alone. Because the mixtures were established in 2022, the comparison between 2023 and 2024 also reflects the transition from the first to the second production year. Therefore, part of the year effect may be related to annual growing conditions, mixture development, differential persistence of component species, and the resulting shifts in botanical composition.

Changes in dry matter yield were associated with a shift in botanical composition as grasses decreased and legumes increased. These changes are important because the relative contribution of grasses and legumes strongly influences biomass formation in mixtures. The contribution of legumes to overall productivity also depends on species proportion and environmental conditions [9,25]. Consistent with this pattern, the observed increase in legumes in 2024 may reflect contrasting responses of grasses and legumes to seasonal growing conditions. The 2024 growing period was characterized by reduced precipitation and elevated temperatures, which may have increased moisture limitation, particularly for grasses. As moisture stress can differentially affect grasses and legumes, it may alter the functional composition within mixtures [29]. According to Hofer et al. [10], legumes may exhibit greater resistance than non-legumes under severe drought, maintaining higher relative biomass compared with grasses. In this context, the increased proportion of legumes likely reflects a relative persistence of the legume component under less favorable growing conditions rather than a compensatory increase in biomass production. However, a higher proportion of legumes does not necessarily compensate for reductions in grass-derived biomass, as grasses typically account for a substantial share of productivity in mixtures [30]. Since botanical composition was not directly tested against environmental variables, this interpretation should be considered an indirect inference based on the observed year-to-year shifts in botanical composition and recorded meteorological conditions.

Chemical composition traits also differed between the two study years, but the expression of mixture type differences was year-dependent. In 2023, mixture types were clearly differentiated, with grazing mixtures showing higher CP, WSC, and DMD and lower fiber fractions than forage and universal mixtures. In 2024, mixture type differences largely converged for CP, CF, MADF, NDF, and WSC, whereas DMD remained different among mixture types. This indicates that annual growing conditions and shifts in botanical composition affected not only biomass production but also the expression of nutritive value differences among mixture types. Importantly, beyond weather-related variation and species composition, nitrogen fertilization represents a key driver of forage quality. It can influence fiber fractions (NDF, ADF), crude protein, and water-soluble carbohydrate concentrations, with effects depending on mixture composition and management intensity [31]. Nitrogen inputs may further modify forage quality indirectly by promoting grass dominance within mixtures, thereby altering their structural and nutritive characteristics. This pattern is consistent with previous studies showing that forage quality responses to climatic factors are often mediated by shifts in species composition, phenology, and plant functional characteristics, whereas productivity may respond more strongly to environmental variability and management [21,22]. The increased legume proportion observed in 2024 may have contributed to the generally higher and more uniform crude protein concentrations, while the strong reduction in WSC indicates that chemical composition was also responsive to year-specific growing conditions. Increased legume presence did not enhance differentiation in chemical composition among mixture types, suggesting that under the conditions observed in 2024, environmental and stand-development effects may have constrained the expression of compositional effects on chemical composition traits.

Taken together, these results show that the contrast between the two study years strongly influenced first-cut mixture performance. Mixtures differing in composition and intended use can effectively target productivity or forage quality under favorable conditions, but their relative performance became less distinct under the contrasting conditions observed in 2024. This suggests that strategies focused primarily on maximizing first-cut biomass may be more vulnerable to year-specific growing conditions. In contrast, mixtures oriented towards forage quality, particularly those incorporating legumes such as white clover, may support higher nutritive value under favorable conditions, although quality-related differences were less consistently expressed between the two years. Because this study was based on two growing seasons and first-cut only, the results should be interpreted as evidence of year-to-year variation in first-cut performance rather than long-term yield stability or climatic resilience. These findings suggest that optimizing grassland mixtures requires balancing productivity and nutritive value, while longer-term and multi-cut studies are needed to evaluate stability across broader climatic conditions. 

## 4. Materials and Methods

### 4.1. Experimental Site and Design

The study was conducted in Dotnuva, Lithuania (55°23′ N, 23°57′ E) during 2023–2024. The soil is classified as *Endocalcari-Epihypogleyic Cambisol* (*CMg-p-w-can*), moderately alkaline (pH 7.7), with 2.05% humus, high in phosphorus (270 mg kg^−1^) and potassium (195 mg kg^−1^), and medium-textured loam. The experimental site comprised 27 grass–legume mixtures, which were sown in June 2022 and arranged in a randomized complete block design with three replications. The plots were 8.25 m^2^ in size. Before sowing, basal N-P-K fertilization was applied at rates of 10-120-180 kg ha^−1^. At the beginning of each vegetative season, 40 kg N ha^−1^ was applied, and an additional 30 kg N ha^−1^ was applied after each cut, except after the final cut. The mixtures were defined according to three main factors: (i) the dominant legume species in the mixture (red clover, lucerne, birdsfoot trefoil), (ii) the proportion of that legume species (40%, 50%, or 60%), and (iii) the mixture type (forage, universal, or grazing). Together, these factors formed a 3 × 3 × 3 treatment structure of dominant legume species, target legume proportion, and mixture type, resulting in 27 mixtures. The 40%, 50%, and 60% legume proportions were calculated based on the recommended monoculture sowing rate of the respective legume species. In this study, mixture types reflected differences in botanical composition and intended use, rather than different management practices applied during the experiment. Each mixture consisted of three or four species, represented by productive cultivars and breeding lines of Lithuanian origin. The legume component included lucerne (*Medicago sativa* L., cv. Malvina), red clover (*Trifolium pratense* L., cv. Arimaičiai), white clover (*Trifolium repens* L., cv. Nemuniai), and birdsfoot trefoil (*Lotus corniculatus* L., breeding line). The grass component comprised perennial ryegrass (*Lolium perenne* L., cv. Elena DS), *×Festulolium* (cv. Punia DS), meadow fescue (*Festuca pratensis* Huds., cv. Alanta), timothy (*Phleum pratense* L., cv. Žolis), and tall fescue (*Festuca arundinacea* Schreb., cv. Monas). The full composition of each mixture is presented in Table 2.

### 4.2. Meteorological Conditions

**Figure 5:**
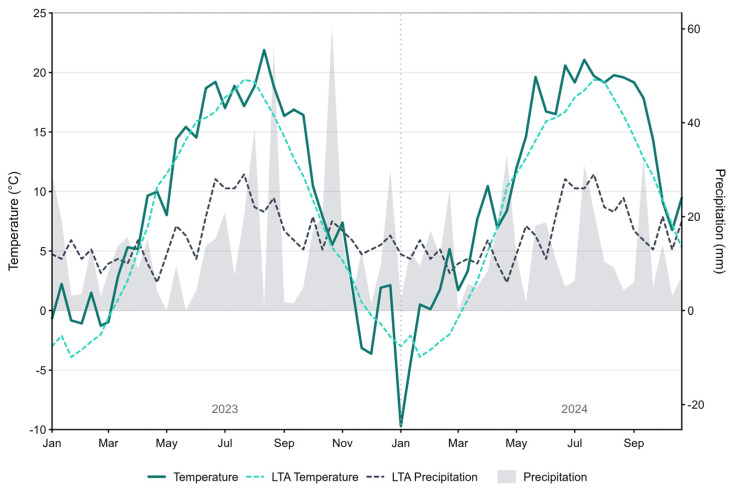
Decadal mean temperature and precipitation, compared with long-term averages, 2023–2024.

Meteorological conditions, including temperature and precipitation in 2023 and 2024, as well as long-term climatic averages, are presented as decadal means (Figure 5) (data from the Lithuanian Hydrometeorological Service). In 2023, air temperatures consistently exceeded the long-term climatological mean, with winter anomalies reaching up to +4 °C, resulting in unstable snow cover (5–11 cm) and generally shallow soil frost of only 5–12 cm. Precipitation patterns were highly uneven: January, August, October, and December received 200–250% of the monthly norms, whereas May and September accumulated only 30–40% of typical values. Annual temperature extremes ranged from −14.1 °C (10 March) to +33.5 °C (16 August), reflecting intensified contrasts between *cold spells* and heat-wave events. Early spring onset, already evident in March, coincided with rapid soil warming (up to +6 °C) and the beginning of vegetation, while autumn was marked by an anomalously warm September (+5 °C above normal). In 2024, nearly all months, except January, were 1–6 °C warmer than the long-term average, while summer and early autumn temperature extremes reached 32–33 °C. Precipitation distribution was highly uneven: February and July received 3–3.5 times the normal amounts, whereas March, May, June, August, and October were distinctly dry, accumulating only 50–70% of typical monthly norms. The onset of winter was marked by sharp contrasts, with temperatures dropping to −24.3 °C in early January, followed by an exceptionally warm February (+2.3 °C on average), during which snow cover persisted only for a few days. Spring began unusually early, as soil temperatures rose to +10 °C already in March, and both April and May were characterized by accelerated vegetation development alongside occasional frost events.

For the early vegetation period relevant to the first cut, the two study years differed in both temperature and precipitation patterns relative to long-term averages. In 2023, active vegetation began earlier than usual, and May was particularly dry, with only 9.6 mm of precipitation, corresponding to about 30% of the monthly norm. In 2024, spring was unusually warm, with March and May being 2.9 °C and 4.1 °C warmer than the long-term averages, respectively; precipitation in these months reached only about 50% and 70% of the monthly norms. 

### 4.3. Dry Matter Yield and Botanical Composition

The plots were harvested multiple times during the growing season; however, the present study focuses only on the first cut. The first cut was conducted at the end of May in both experimental years (2023 and 2024), when grasses were at the heading stage, and legumes were at the beginning of flowering. Harvesting was performed with a self-propelled hay mower at an approximate cutting height of 5 cm above ground, and biomass mass was determined directly in the mower bunker. Dry matter productivity of the mixtures was assessed by taking a fresh herbage sample of 0.5 kg, which was dried to a constant weight at 105 °C in a well-ventilated drying oven [32]. Dry matter yield per hectare was then calculated based on the dry weight of each sample. The botanical composition of the mixtures was determined during the first cut. From each plot, a 0.5 kg fresh herbage sample was collected and manually separated into three botanical groups according to plant families: legumes, grasses, and forbs (other non-sown species). Each group was weighed individually, and its relative proportion in the mixture was calculated and expressed as a percentage of the total sample weight.

### 4.4. Chemical Composition Analysis

For chemical analyses, samples were collected prior to harvest using the same sampling procedure as for yield determination. Fresh material was thoroughly mixed and cut into 3–5 cm fragments. The samples were initially fixed at 105 °C for 20 min and subsequently dried at 65 ± 5 °C in a well-ventilated drying chamber until constant weight. The dried material was then homogenized and finely ground using a Retsch™ ZM 200 ultra-centrifugal mill. Forage chemical composition analyses were performed in the laboratory using standardized analytical procedures. Near-infrared reflectance spectroscopy (NIRS) was used for the routine determination of crude protein (CP), crude fiber (CF), modified acid detergent fiber (MADF), neutral detergent fiber (NDF), water-soluble carbohydrates (WSC), and dry matter digestibility (DMD), based on calibration models routinely applied and validated by the laboratory for forage samples [33]. Crude protein concentration was additionally determined using the Kjeldahl method, while water-soluble carbohydrate concentration was determined by a standard spectrophotometric method. These reference analyses were used to provide an additional check of the reliability of the forage quality assessment.

### 4.5. Statistical Analysis

The obtained data were statistically processed using R software (version 4.3.1; R Core Team, Vienna, Austria). Prior to modeling, data were checked for normality (Shapiro–Wilk test) and homogeneity of variances (Levene’s test). Dry matter yield was analyzed using a linear mixed-effects model. Legume proportion, mixture type, year, and legume species were treated as fixed effects. The interaction among legume proportion, mixture type, and year was included in the model, while legume species was included as a main effect. Block/replication and plot identity were included as random effects to account for the randomized complete block design and repeated observations of the same plots across years. Fixed effects were evaluated using model-based ANOVA tests. Where relevant, estimated marginal means were used to interpret mixture type effects within each year, with Tukey-adjusted pairwise comparisons. In 2024, data from three individual plots were unavailable due to sample loss; these observations were treated as missing values, and all models were fitted using the available data without imputation. Chemical composition traits (CP, CF, MADF, NDF, WSC, DMD; all expressed as % DM) were analyzed using linear mixed-effects models. For each trait, mixture type, year, and the mixture type × year interaction were treated as fixed effects, while block/replication and plot identity were included as random effects. Fixed effects were evaluated using model-based ANOVA tests. Estimated marginal means (EMMeans) and standard errors were obtained using the emmeans package. Mixture-type effects were interpreted within each year using Tukey-adjusted pairwise comparisons. Results are presented as EMMeans ± SE, with different letters indicating statistically distinct groups. Descriptive statistics (mean, SD, SE, 95% CI, minimum, maximum) were calculated to characterize variability within treatments. To explore multivariate relationships among agronomic and chemical composition traits, PCA was performed separately for each year using plot-level observations. The analysis included dry matter yield, chemical composition traits, and plant height. PCA was used as an exploratory multivariate approach to describe trait relationships and visual separation among mixture types; no formal statistical test was applied to compare PCA group separation between years. PCA results were visualized as biplots with 95% confidence ellipses for each mixture type (forage, grazing, universal). PCA was carried out using the packages FactoMineR, factoextra, and ggplot2. 

## 5. Conclusions

Year-to-year variation between the two study years was the main factor shaping first-cut performance of grass–legume mixtures, often overriding differences among mixture types. The strong decline in dry matter yield in 2024, together with the convergence of most chemical composition traits among mixture types, indicates that annual growing conditions affected both productivity and nutritive value expression. Under the more favorable conditions of 2023, mixture type differences were clearly expressed, whereas in 2024 they became less distinct, except for DMD. From a practical perspective, mixture selection should consider both the intended production goal and the expected variability in growing conditions. Forage mixtures may be more suitable when maximizing first-cut biomass is the main objective under favorable conditions, whereas grazing mixtures may provide a more balanced option when maintaining nutritive value is important. These results should be interpreted within the context of two growing seasons and first-cut data only.

## Figures and Tables

**Table 1:** Effect of mixture type on chemical composition in 2023 and 2024 (EMMeans ± SE, % DM).

Indicator	Year	Forage	Universal	Grazing	Significance
CP	2023	11.1 ± 0.4 ^b^	11.4 ± 0.4 ^b^	15.1 ± 0.4 ^a^	*p* < 0.0001
CF	2023	29.1 ± 0.4 ^a^	29.5 ± 0.4 ^a^	23.3 ± 0.4 ^b^	*p* < 0.0001
MADF	2023	26.5 ± 0.3 ^a^	26.7 ± 0.3 ^a^	20.9 ± 0.3 ^b^	*p* < 0.0001
NDF	2023	50.3 ± 0.6 ^a^	50.3 ± 0.6 ^a^	41.2 ± 0.6 ^b^	*p* < 0.0001
WSC	2023	62.9 ± 0.7 ^b^	61.2 ± 0.7 ^b^	74.8 ± 0.7 ^a^	*p* < 0.0001
DMD	2023	20.6 ± 0.8 ^a^^b^	19.5 ± 0.8 ^b^	23.2 ± 0.8 ^a^	*p* = 0.0085
CP	2024	15.5 ± 0.4 ^a^	16.2 ± 0.4 ^a^	16.5 ± 0.4 ^a^	n.s.
CF	2024	25.9 ± 0.4 ^a^	25.3 ± 0.4 ^a^	25.8 ± 0.4 ^a^	n.s.
MADF	2024	23.0 ± 0.3 ^a^	22.9 ± 0.3 ^a^	23.6 ± 0.3 ^a^	n.s.
NDF	2024	43.8 ± 0.6 ^a^	43.0 ± 0.6 ^a^	45.1 ± 0.6 ^a^	n.s.
WSC	2024	17.1 ± 0.7 ^a^	17.7 ± 0.7 ^a^	18.1 ± 0.7 ^a^	n.s.
DMD	2024	66.8 ± 0.8 ^b^	69.1 ± 0.8 ^a^^b^	69.8 ± 0.8 ^a^	*p* = 0.0330

Means within a row followed by different letters differ significantly between forage, universal, and grazing mixtures (*p* < 0.05). Abbreviations: CP—crude protein; CF—crude fiber; MADF—modified acid detergent fiber; NDF—neutral detergent fiber; WSC—water-soluble carbohydrates; DMD—dry matter digestibility; n.s.—not significant.

**Table 2:** Composition of grass–legume mixtures (n = 27).

Major Component of the Mixtures	Grasses							Legume ^†^
	Pr + Mf		Tf + F + T			Tf + F		+ Wc
L (40/50/60%)	#	#	*	*	*	&	&	&
Rc (40/50/60%)	#	#	*	*	*	&	&	&
Bt (40/50/60%)	#	#	*	*	*	&	&	

Symbols: #—forage mixtures, *—universal mixtures, &—grazing mixtures. Each mixture contained 40, 50, and 60% of the respective legume component, with the remaining proportion consisting of grasses and, where indicated, white clover. Abbreviations: L—lucerne, Rc—red clover, Bt—birdsfoot trefoil, Wc—white clover, Pr—perennial ryegrass, Mf—meadow fescue, Tf—tall fescue, F—*×Festulolium*, T—timothy. † White clover (Wc) was included only in grazing mixtures with lucerne (L) and red clover (Rc).

## Data Availability

The data presented in this study are available on request from the corresponding author. The data are not publicly available due to privacy and ethical restrictions.
